# Effects of Sulfuric Acid on the Curing Behavior and Bonding Performance of Tannin–Sucrose Adhesive

**DOI:** 10.3390/polym10060651

**Published:** 2018-06-11

**Authors:** Zhongyuan Zhao, Yanfeng Miao, Ziqian Yang, Hua Wang, Ruijuan Sang, Yanchun Fu, Caoxing Huang, Zhihui Wu, Min Zhang, Shijing Sun, Kenji Umemura, Qiang Yong

**Affiliations:** 1 Co-Innovation Center of Efficient Processing and Utilization of Forest Resources, Nanjing Forestry University, Nanjing 210037, China; 2 College of Furnishings and Industrial Design, Nanjing Forestry University, Nanjing 210037, China; Myf1203@163.com (Y.M.); Yangziqian@njfu.edu.cn (Z.Y.); Myhuae@163.com (H.W.); Sangruijuan@njfu.edu.cn (R.S.); Tracy_528@126.com (Y.F.); Wzh550@sina.com (Z.W.); 3 College of Chemical Engineering, Nanjing Forestry University, Nanjing 210037, China; Szxapy@163.com; 4 Laboratory of Sustainable Materials, Research Institute for Sustainable Humanosphere, Kyoto Univeersity, Gokasho, Uji, Kyoto 611-0011, Japan; Zhang888@rish.kyoto-u.ac.jp; 5 College of Material Science and Engineering, Nanjing Forestry University, Nanjing 210037, China

**Keywords:** natural adhesive, tannin, sucrose, sulfuric acid catalyst, curing behavior, particleboard

## Abstract

The development of biomaterials-based adhesives is one of the main research directions for the wood-based material industry. In previous research, tannin and sucrose were used as adhesive to manufacture particleboard. However, the reaction conditions need to be optimized. In this study, sulfuric acid was added to the tannin–sucrose adhesive as a catalyst to improve the curing process. Thermal analysis, insoluble mass proportion, FT-IR, and solid state ^13^C NMR were used to investigate the effects of sulfuric acid on the curing behavior of tannin and sucrose. Thermal analysis showed weight loss and endotherm temperature reduced from 205 and 215 to 136 and 138 °C, respectively, by adding sulfuric acid. In case of the adhesive with pH = 1.0, the insoluble mass proportion achieved 81% at 160 °C, which was higher than the reference at 220 °C. FT-IR analysis of the uncured adhesives showed that adding sulfuric acid leads to hydrolysis of sucrose; then, glucose and fructose converted to 5-hydroxymehthylfurfural (HMF) and levulinic acid. Dimethylene ether bridges were observed by FT-IR analysis of the cured adhesives. The results of solid state ^13^C NMR spectrum indicated that 5-HMF participated in the curing process and formed methylene bridges with the C8 position of the resorcinol A-rings of tannin, whereas dimethylene ether bridges were detected as a major chemical chain of the polymer. Lab particleboards were produced using 20 wt % resin content at 180 °C and 10 min press time; the tannin–sucrose adhesive modified with sulfuric acid to pH = 1.0 exhibited better performance than the unmodified tannin–sucrose adhesive; the properties of the boards fulfilled the requirement of Japanese Industrial Standard (JIS) A5908 type 15.

## 1. Introduction

Wood-based materials, such as particleboard, plywood, or fiberboard, are generally used in housing construction, interior decoration, and furniture manufacturing, which are frequently present in living environments [1,2]. Generally, these materials are manufactured by bonding the wooden elements with synthetic resins such as formaldehyde-based, isocyanate-based, polyvinyl acetate adhesives, and so on [3,4,5]. However, as a result of concerns about formaldehyde emissions and dependency on fossil resources, it is necessary to develop adhesives based on renewable resources, such as lignin, soybean, starch, or tannin [6,7,8,9]. However, because of the generally weak bonding performance of biomass resources, most of the studies focus on replacing only a certain part of the chemical substances derived from fossil resources with these bio-materials, which does not solve the issues fundamentally. Therefore, a significant research direction on natural adhesive aims to utilize renewable resources as only raw materials when synthesizing high-performance adhesive [10,11,12]. 

Condensed tannins comprise 90% of the total world production of commercial tannins (200,000 tons per year) [13]. Tannins are widely distributed in nature, especially in the wood and bark of various trees, such as *Acacia* (wattle or mimosa bark extract), *Schinopsis* (quebracho wood extract), *Tsuga* (hemlock bark extract), and *Rhus* (sumach extract) [9]. Condensed tannins contain high amounts of polyphenols, which commonly are utilized to produce leather [14,15]. The monomer of wattle tannin contains resorcinol A-rings and catechol or pyrogallol B-rings, and the free C6 or C8 sites on the A-ring react with active substances because of their strong nucleophilicity [16]. An example of such an active substance is sucrose, which is a common disaccharide, and 5-hydroxymehthylfurfural (5-HMF) generates by heat treatment [17,18].

Considering the characteristics of chemical properties, tannin and sucrose were chosen as adhesive components for the work reported here. In a previous work, lab particleboards with 9 mm thickness were produced at a hot pressing temperature of 220 °C and hot pressing time of 10 min; the mechanical properties and water resistance of the board fulfilled the requirements of Japanese Industrial Standard (JIS) A 5908 type 18 standard (2003) [19,20]. However, the reaction conditions needed for the reaction between tannin and sucrose had been very harsh; therefore, it was requisite to develop a method that can reduce the curing temperature or time.

Judging from the results of the curing mechanism and thermal analysis of tannin–sucrose adhesives, 5-HMF is generated at 200 °C and then chemically reacts with tannin. The insoluble mass proportion was 0% when the temperature was lower than 200 °C, indicating there was not any reaction happening between tannin and sucrose [20]. Hence, the high curing temperature between tannin and sucrose is a result of the generation temperature of 5-HMF. Based on some studies, inorganic acids could be used as catalyst to gain 5-HMF from sucrose already at a lower temperature [21,22,23]; this also could cause hydrolysis of tannin [24], which possibly also will accelerate the curing reaction rate. In this study, sulfuric acid was used as a catalyst to reduce the reaction temperature between tannin and sucrose: the effects of the sulfuric acid addition on the curing temperature, the curing behavior, and the bonding performance were investigated. 

## 2. Materials and Methods

### 2.1. Materials

Wattle tannin (commercial name: tannic acid ME) was purchased from the Fuji Chemical Industry Co. (Wakayama, Japan). Sucrose (guaranteed reagent) and sulfuric acid were purchased from Nacalai Tesque, Inc. (Kyoto, Japan) and Wako pure chemical industries Ltd. (Kyoto, Japan), respectively. Tannin and sucrose were used without further purification, but were dried in a vacuum oven at 60 °C for 15 h. The wood particles were screened by a sieving machine to collect particle sizes in the range of 0.9 to 5.9 mm. Before particleboard manufacturing, the particles (original moisture content 3‒4 wt %) were dried in an oven at 80 °C for 12 h to a final moisture content of 2 wt %.

### 2.2. pH Value Adjustment of Adhesive Solution by Adding Sulfuric Acid

The sulfuric acid content in the tannin–sucrose mixture was determined by the adjustment of pH values. The optimal ratio of tannin to sucrose is 25:75 [19]. In this study, 25 g tannin and 75 g sucrose were mixed in a beaker, and 150 g distilled water was added to the mixture to get a tannin–sucrose adhesive solution at 40 wt % concentration. Pure sulfuric acid was also mixed with distilled water to 40 wt % concentration. Subsequently, 0.1 g of the sulfuric acid solution was added stepwise to the tannin–sucrose solution. The viscosity and pH of the solution at 20 °C was measured by a rotational viscometer (Viscolead One, Fungilab S.A., Barcelona, Spain) and a pH meter (D-51, Horiba Scientific, Kyoto, Japan), respectively. This process was repeated several times until the tannin–sucrose solution reached pH 1.0. Basic information regarding the tannin–sucrose–sulfuric acid mixture is presented in Table 1.

### 2.3. Thermal Analysis

After the various pH adjustments, 100 g of each variation in sulfuric acid content was poured into glass vials and then freeze-dried. The dried mixtures were pulverized to smaller than 250-μm mesh size to obtain the uncured adhesive powder. Thermogravimetric analysis (TGA) and differential scanning calorimetry (DSC) were carried out using a TGA 2050 (TA Instruments, Tokyo, Japan) and DSC 2910 (TA Instruments, Tokyo, Japan), respectively, and the samples were scanned from room temperature to 400 °C at a rate of 10 °C/min under nitrogen purging with the flow rate at 100 and 40 mL/min, respectively.

### 2.4. Measurement of Insoluble Mass Proportion

Samples of the uncured adhesive powders were separately heated up to 160, 180, 200, and 220 °C, respectively, for 10 min to achieve curing. Then, 2 g samples of each cured adhesive sample were boiled in distilled water for 4 h to obtain the wet insoluble residue. The boiling treatment was carried out in triplicate. All samples obtained from heating treatments, as well as the residues after the boiling treatment, were vacuum-dried at 60 °C for 15 h. The insoluble mass proportion was calculated by the following equation:

(1) $$\mathrm{Insoluble}\;\mathrm{mass}\;\mathrm{proportion}\left(\%\right)=\frac{\mathrm{Weight}\left(\mathrm{dried}\;\mathrm{insoluble}\;\mathrm{mass}\right)}{\mathrm{Weight}\left(\mathrm{cured}\;\mathrm{adhesive}\right)}\times 100\%$$

### 2.5. Fourier Transform Infrared Spectra (FTIR)

The chemical changes between uncured and cured adhesives were investigated by FTIR spectra using spectrophotometer FT/IR-4200 (JASCO Corporation, Easton, MD, USA), with KBr disk method and recording with an average of 32 scans at a resolution of 4 cm^−1^. The two adhesives, (i) adjusted to pH = 1.0 and heated at 160 °C for 10 min and (ii) at original pH = 4.8 heated at 220 °C for 10 min, gave the same insoluble mass proportion; both kinds of the insoluble mass were also investigated by FT-IR for the effects of sulfuric acid on the reaction mechanism.

### 2.6. ^13^C Cross Polarization-Magic Angle Spinning (CP-MAS) NMR

^13^C CP–MAS NMR spectra were acquired at room temperature on a Varian 400 NMR system spectrometer with a Varian 5 mm CP–MAS and a multipulse probe. Powder samples were placed in Si_3_N_4_ rotors 5 mm in diameter with an o-ring cap, and then spun at the magic angle at the frequency of 8–9 kHz. ^13^C NMR spectra were taken at 100.56 MHz with a 40 ms acquisition period, 30.5 kHz spectral width, and a frequency of 86 kHz. The pulse sequence was continuous ^1^H decoupling with a small phase incremental alteration (SPINAL). Cross-polarization involved 2.0 ms contact time. The ^13^C CP–MAS NMR spectra were obtained using the regular CP–MAS sequence at a 500 μs contact time with a 4.0 μs long π/2 pulse and a 5.0 s of recycle delay.

### 2.7. Manufacture of Particleboard

The two adhesives at pH = 4.8 and 1.0 with 40 wt % concentration were sprayed onto wood particles in a blender at 20 wt % resin content based on the weight of the oven-dried particles, and the sprayed particles were again dried at 80 °C for 12 h until the moisture content was 4–7 wt %. The dried particles were mat-formed using a forming box of 300 mm× 300 mm. Then, the mat was hot-pressed at 180 °C for 10 min with distance bars of 9 mm to control the thickness. The target density of the boards was 800 kg/m^3^. Considering the given bonding properties of natural substances, the resin content and the hot pressing time were adjusted in this stage of the work still to significantly higher values than usually given for synthetic resins in the wood industry.

### 2.8. Evaluation of Particleboard Properties

The particleboards were conditioned for 1 week at 20 °C and 60% relative humidity (RH), and then evaluated according to the Japanese Industrial Standard for particleboard (JIS A 5908, 2003). The static three-point bending test was carried out on a 200 mm × 30 mm × 9 mm specimen from each board, and the effective span and loading speed were 150 mm and 10 mm/min, respectively. The modulus of rupture (MOR) was calculated from the bending test. The internal bond strength (IB) test was performed on a 50 mm × 50 mm specimen with a loading speed of 2 mm/min, and thickness swelling (TS) after water immersion for 24 h at 20 °C was measured in specimens of the same size. Each experiment was performed five times, and the average values and standard deviations were calculated. Statistical significance was considered for *p*-values < 0.5.

## 3. Results and Discussion

### 3.1. Thermal Analysis

**Figure 1:**
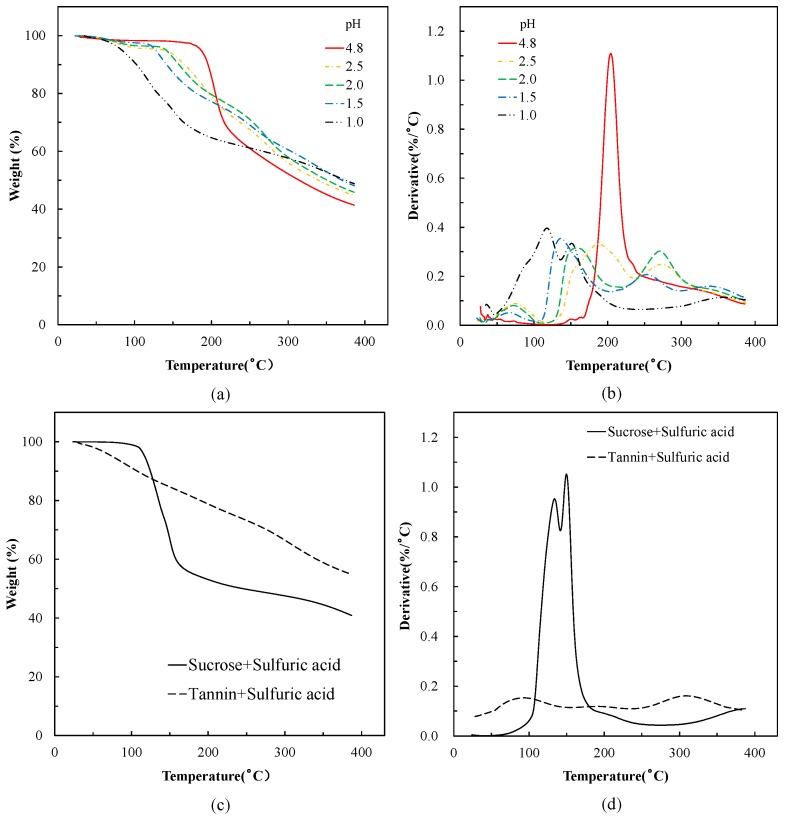
Thermogravimetric (TG) and derivative TG (DTG) curves of mixtures. (**a**) TG curves of tannin–sucrose–sulfuric acid mixture with different pH; (**b**) DTG curves of tannin–sucrose–sulfuric acid mixture with different pH; (**c**) TG curves of tannin–sulfuric acid and sucrose–sulfuric acid mixture at pH = 1.0; (**d**) DTG curves of tannin–sulfuric acid and sucrose–sulfuric acid mixture at pH = 1.0.

Figure 1 shows the thermogravimetric (TG) and derivative TG (DTG) curves of the various mixtures at the different pH adjustments. Tannin and sucrose, both separately adjusted with sulfuric acid to pH = 1.0 were used as control. The acidified tannin exhibited a linear profile, indicating a continuous degradation during heating. Contrary to this, the acidified sucrose showed a two-step degradation in the DTG curve with two peaks at 138 and 155 °C; these mass losses seem to be attributed to the pyrolysis or caramelization of sucrose in acidic conditions [25]. The DTG curve of the unmodified tannin/sucrose mixture (pH 4.8) shows a sharp peak at around 205 °C, which was considered as the thermolysis of sucrose, generation of 5-HMF, and reaction with tannin [20]. The addition of sulfuric acid to the tannin–sucrose mixes reduces the temperature of mass loss, as seen from both curves, TG and DTG. The version with pH = 1.0 exhibits a two-step degradation; the first stage occurs at 50 to 115 °C, based on the DTG curve of acidified tannin, this was possible because of the degradation of tannin. The second peak was observed at around 153 °C, which seems to be caused by the pyrolysis or caramelization of sucrose [26,27]. Moreover, additional peaks in the range 254 to 274 °C were observed for the samples with pH even higher; with decreasing pH values, the height of these peaks became less and finally the peak disappeared in the pH = 1.0 adhesive. Judging from the DTG profile of sucrose in the previous research, this was possible because of the production of a black aerated char-like solid [20]; the addition of bigger amounts of sulfuric acid obviously changed the reaction equilibrium leading to excess sucrose formation in the reaction system.

**Figure 2:**
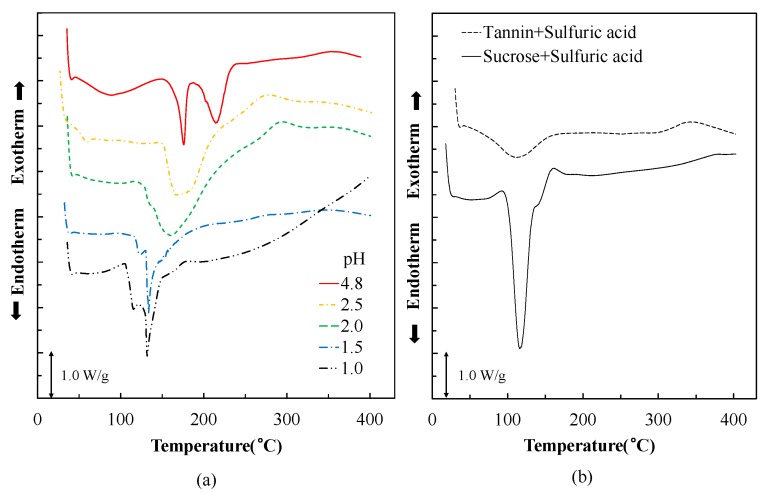
Differential scanning calorimetry (DSC) curves of the mixtures. (**a**) TG curves of tannin–sucrose–sulfuric acid mixture with different pH; (**b**) TG curves of tannin–sulfuric acid and sucrose–sulfuric acid mixture at pH = 1.0.

Figure 2 shows DSC curves of the adhesives. The mixture of sucrose and sulfuric acid has a sharp endothermic peak at 118 °C, indicating melting and caramelization already at lower temperatures [20]. The acidified tannin exhibits only a weak endotherm peak at around 115 °C, attributed to the degradation of tannin [24]. The tannin–sucrose mix at pH = 4.8, without addition of sulfuric acid, gives two endothermic peaks at 180 and 215 °C, which were attributed to the melting of sucrose and the curing reaction, respectively [20]. The addition of sulfuric acid to the tannin–sucrose mix merges the two endotherm peaks to one peak and moves this to lower temperatures. At pH = 1.0 and 1.5, a shoulder appeared at around 115 °C, which is possible because of the decomposition of tannin or the melting of sucrose. In addition, a sharp peak located at 128 °C was attributed to the caramelization of sucrose or the reaction between 5-HMF and tannin [26,27]. Judging from the results of thermal analysis, the addition of sulfuric acid significantly reduced the reaction temperature between tannin and 5-HMF, which was produced from heating of sucrose.

### 3.2. Insoluble Mass Proportion

**Figure 3:**
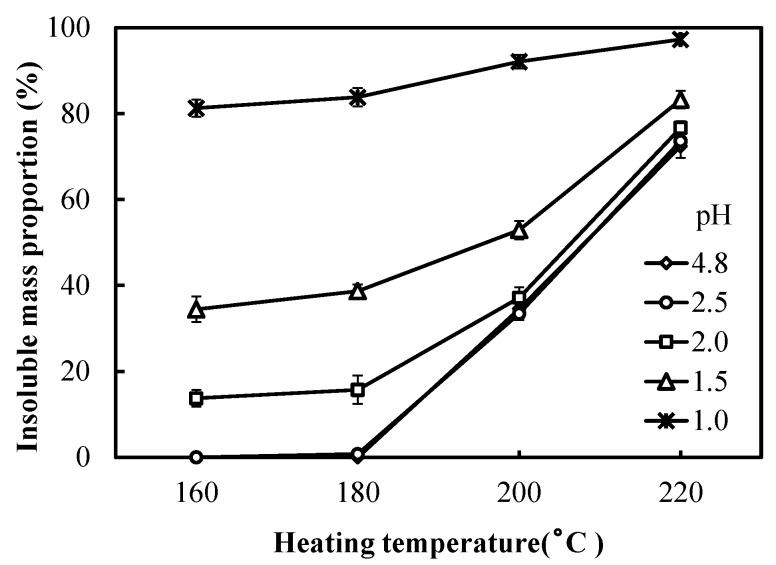
Insoluble mass proportion of the adhesives with different pH values and heated for 10 min.

The increase of insoluble mass proportion of the various adhesives when heating to different temperatures is shown in Figure 3. The adhesives adjusted to different pH values were heated at 160, 180, 200, and 220 °C for 10 min, and then the partly cured adhesives were boiled for 4 h before determining the insoluble mass proportion. Variance analysis (ANOVA) showed no significant (*p* > 0.05) difference between the adhesives with 4.8 and 2.5 pH in all heating temperature conditions. The higher the temperatures, the higher was the achieved insoluble mass proportion of all mixes. The unmodified tannin–sucrose mixture, which adjusted to pH = 2.5, showed no development of insoluble material at the two lower temperatures of 160 and 180 °C. This could be verified by the results of thermal analysis showing that the mass loss and endothermic reaction peaks of these mixtures were located at around 190–200 °C; at decreasing pH values, the insoluble mass proportion increased for each temperature level. The highest insoluble mass proportion was obtained from the adhesive at pH = 1.0; at 160 °C, the ratio was already 81%; this was higher than most other adhesives treated at 220 °C and indicates the strong curing reaction between tannin and 5-HMF (created by sucrose). This reflects the fact that the addition of sulfuric acid could reduce the curing temperature of the acidified tannin–sucrose mixes.

### 3.3. FT-IR Spectroscopic Analysis

**Figure 4:**
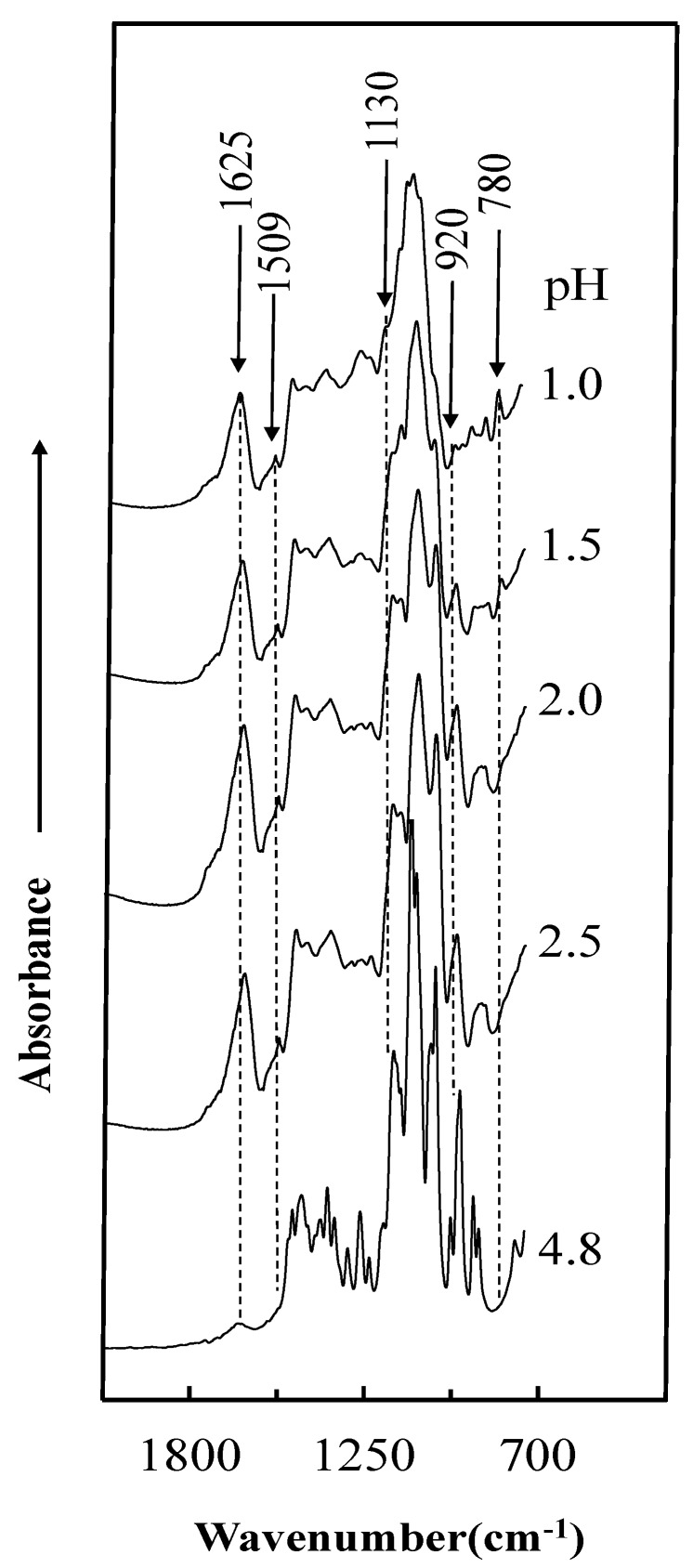
FT-IR spectra of uncured adhesives.

The FT-IR curves of the various uncured acidified tannin–sucrose adhesives (Figure 4) show two absorption peaks (1130 and 920 cm^−1^), disappearing when the pH is reduced. The peak at 1130 cm^−1^ was assigned to glycosidic linkage (C–O–C) of sucrose [28,29], and the absorption band at around 920 cm^−1^ was attributed to the pyranose ring of sucrose [30]. This indicates that hydrolysis happened by adding sulfuric acid into the sucrose solution. In addition, the absorption bands at 1625, 1509, and 780 cm^−1^ increased clearly at increasing addition of sulfuric acid. The peak located at 1625 cm^−1^ typically derives from aromatic C=C stretching, possibly attributed to the dehydration of sucrose or levulinic acid [31]. The absorption band at 1509 and 780 cm^−1^ was a result of the characteristic of C=C stretching vibration and unsubstituted CH=CH of 5-HMF, respectively [32,33]. The results of these spectra changes of uncured adhesives indicate that the addition of sulfuric lead to the hydrolysis of sucrose, and then, glucose and fructose converted to 5-HMF and levulinic acid.

**Figure 5:**
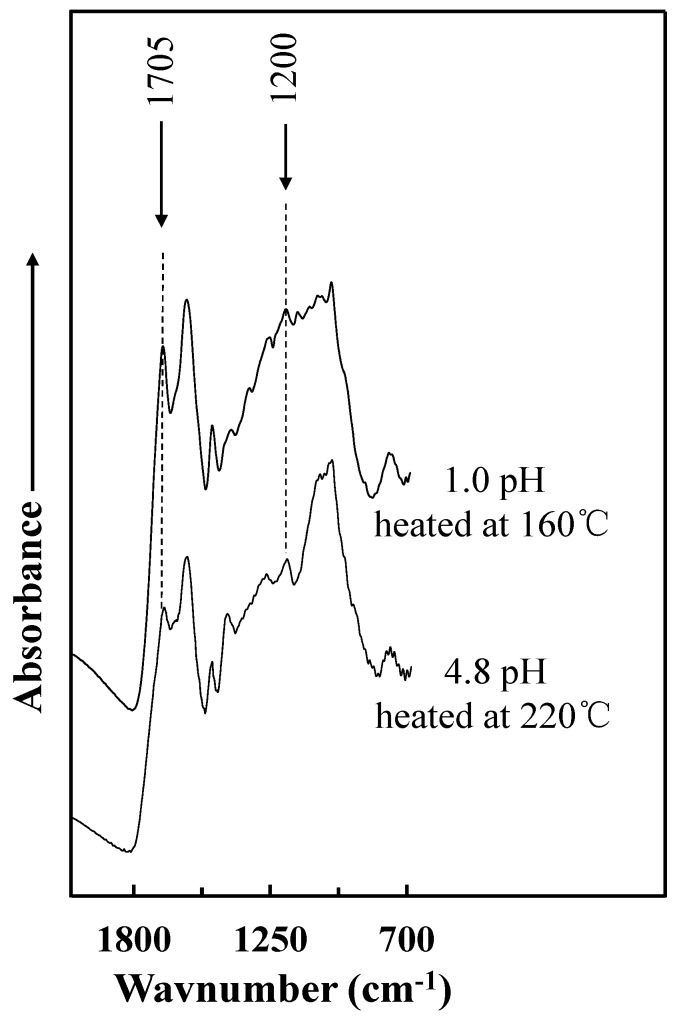
FT-IR spectra of insoluble matter obtained from cured adhesives.

Figure 5 shows the FT-IR profiles of the insoluble mass of the acidified tannin–sucrose adhesive with pH = 1.0 cured at 160 °C for 10 min and the unmodified tannin–sucrose adhesive with pH = 4.8 cured at 220 °C for 10 min. Two peaks increased as pH was reduced: (i) the band at 1705 cm^−1^, attributed to C=O stretching of the carbonyl group [34]; and (ii) the band at 1200 cm^−1^, associated with the –C–O stretching of the benzene nucleus and/or dimethylene ether bridges (–CH_2_–O–CH_2_–) [16]. The increasing of dimethylene ether bridges indicated that the sulfuric acid promotes the curing between tannin and 5-HMF, which was created by the heating of sucrose.

### 3.4. ^13^C CP–MAS NMR Analysis

**Figure 6:**
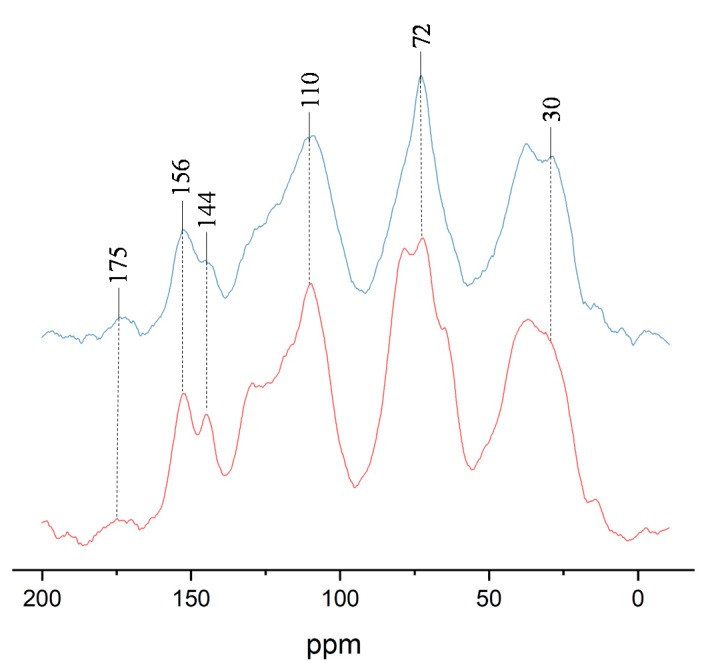
Solid state ^13^C NMR spectrum of the insoluble matter of tannin–sucrose–sulfuric acid adhesive with 1.0 pH cured at 160 °C for 10 min (blue) and tannin–sucrose adhesive cured at 220 °C for 10 min (red).

**Figure 7:**
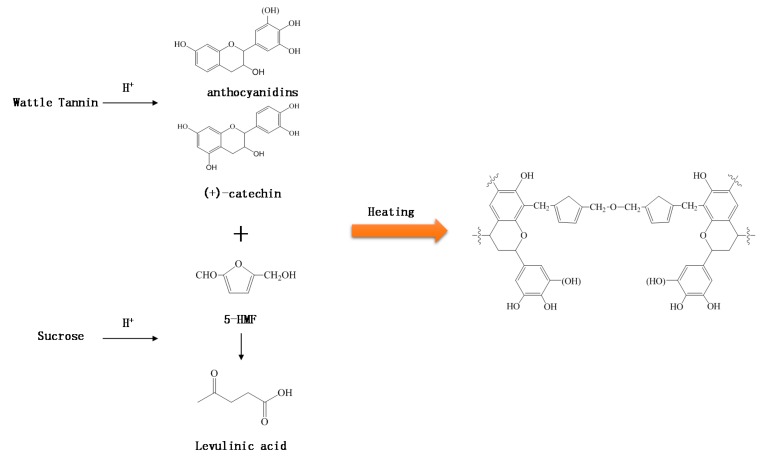
Possible reaction process between wattle tannin and sucrose by adding sulfuric acid.

In Figure 6, the solid state ^13^C NMR spectra of (i) the insoluble matter obtained from the tannin–sucrose–sulfuric acid adhesive with pH = 1.0 pH cured at 160 °C for 10 min, and (ii) the unmodified tannin–sucrose adhesive with pH = 4.8 and cured at 220 °C for 10 min, are compared. The spectra look very similar, which means that the addition of sulfuric acid changed the rate of the curing reaction, but not the reaction mechanisms between tannin and sucrose–5-HMF. The peak at 110 ppm was attributed to the C2 and C3 of the furanic ring of 5-HMF, while the other two signals at 144 and 156 ppm, which overlap with the aromatic C–O of the tannin, certified that 5-HMF participated in the curing reaction [35]. In addition, the peak at around 110 ppm was also attributed to the C8 position of tannin for methylene ether bridging [36,37]. A small peak at 175 ppm was derived from the carbonyl group, and this peak was possible as a result of the unreacted aldehyde group of 5-HMF [38]. A major peak at around 72 ppm was very intense in both adhesives, which was attributed to the dimethylene ether bridges (–CH_2_–O–CH_2_–) between two 5-HMF molecules [38,39]. The absorption at 30 ppm from the insoluble mass of tannin–sucrose–sulfuric acid adhesive was a result of methylene bridges (–CH_2_–), which were formed by the reaction of 5-HMF with tannin [40]. The results of ^13^C NMR spectrum show that 5-HMF participated in the curing reaction and forms methylene bridges with C8 of the resorcinol A-rings of tannin. Furthermore, dimethylene ether bridges were detected as a major chemical structure of the cured tannin–sucrose mixture. The sulfuric acid played a catalytic role and accelerated the generation of 5-HMF already at lower temperatures, and thus reduces the necessary curing temperature between tannin and sucrose. Based on the chemical information gained from FT-IR and solid state ^13^C NMR, a scheme of the curing process of tannin–sucrose–sulfuric acid is shown in Figure 7.

### 3.5. Bending, Internal Bond Strength, and Thickness Swelling of Particleboard

**Figure 8:**
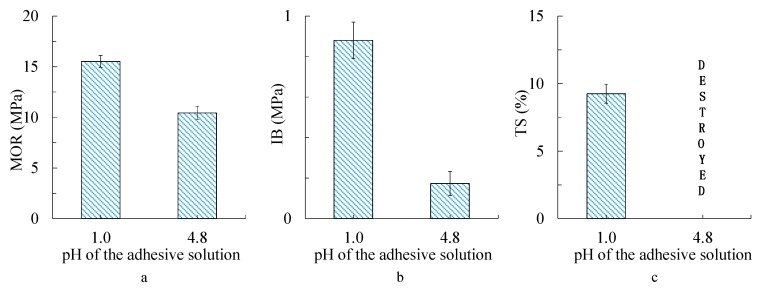
Mechanical properties and water resistance of the particleboards bonded by the tannin–sucrose–sulfuric acid adhesive (pH 1.0) and the tannin–sucrose adhesive (pH 4.8) at 180 °C for 10 min. (**a**) Bending properties; (**b**) internal bond (IB) strength properties; and (**c**) thickness swelling (TS) properties. MOR—modulus of rupture.

Figure 8 shows the mechanical properties (MOR and IB) and the thickness swelling (TS) of lab particleboards with the unmodified tannin–sucrose adhesive (pH = 4.0) and the acidified version (pH = 1.0). MOR, IB, and TS based on the acidified adhesive were improved when compared with the unmodified adhesive; the samples for TS based on the pH = 4.8 version were even destroyed during the water immersion. This indicates that the addition of sulfuric acid improved all properties of the lab boards significantly. The properties of the particleboard bonded by 1.0 pH adhesive fulfill the requirements type 15 of JIS A5908, however, it must be considered that the preparation conditions of the lab boards are still far away from usual industrial conditions. In a previous study, lab particleboards were prepared using the non-modified tannin–sucrose mixture (pH = 4.8) as adhesive at even higher resin content and press temperatures; the boards showed better mechanical properties (MOR 21.4 MPa, IB 1.45 MPa) at more or less the same TS (9.6%) when compared with the acidified version at lower resin content and lower press temperatures. Because of the fact that several parameters are different in this comparison, a clear statement is not possible; however, it can nevertheless be concluded that the sulfuric acid promoted the curing process between tannin and sucrose (via 5-HMF). The low pH of the adhesive mixture may not only induce acidic catalysis of the formation of 5-HMF out of sucrose, but may also lead to a certain decomposition of cellulose and hemicellulose, especially at higher temperatures and long press times [41].

## 4. Conclusions

Sulfuric acid was added to tannin–sucrose mixtures as a catalyst to accelerate the reaction rate of the curing process and to reduce the curing temperature. Thermal analysis shows that, in this reaction, the rate of weight loss increases and temperature decreases the lower the pH of the adhesive solution. Both TG/DTG and DSC showed that at pH = 1.0 pH, the decomposition of sucrose to 5-HMF occurred at much lower temperatures (136 and 128 °C, respectively) when compared with the unmodified tannin–sucrose mixture at pH = 4.8 (205 and 215 °C, respectively). Additionally, the generation of insoluble residues for the low pH version occurred at lower temperatures and to a further extent when compared with the unmodified version.

FT-IR of the uncured adhesives confirmed that the addition of sulfuric acid led to the hydrolysis of sucrose to glucose and fructose, and further to 5-HMF and levulinic acid. Dimethylene ether bridges and carbonyl group were detected by FT-IR analysis of the cured adhesives. Solid state ^13^C NMR indicated that 5-HMF participates in the curing reaction and forms methylene bridges with the C8 of the resorcinol A-rings of tannin; further, dimethylene ether bridges were detected as a major chemical structure in the cured resin, originating from the condensation reaction between the hydroxyl groups of two 5-HMF molecules already linked to tannin. 

The properties of lab particleboards using the acidified tannin–sucrose mix as adhesive improved significantly when compared with the unmodified version. In principle, the requirements for board type 15 of JIS A5908 were fulfilled, however, at production conditions, in terms of adhesive consumption and press time, which are still far outside industrial and even usual lab conditions. The improvement of the board properties is based on the higher rate of thermal decomposition of sucrose to 5-HMF, even at a lower temperature, when the pH of the tannin–sucrose mixture is adjusted to low values. Possible positive or also negative consequences of the low pH on potential decomposition reactions of cellulose or other wood constituents, as well as risk of corrosion, however, need further attention when introducing this type of adhesive into the industrial practice. Based on these considerations, further development work will concentrate on reducing acidity of this type of adhesive; furthermore, possible application for plywood shall be investigated.

## Figures and Tables

**Table 1:** Viscosity and pH of the tannin–sucrose–sulfuric acid solution.

Addition of Sulfuric Acid Solution (g)	Mixture Ratio of Tannin–Sucrose–Sulfuric Acid	Concentration (wt %)	Viscosity at 20 °C (mPa·s)	pH
0	25:75:0	40	51.3	4.8
0.9	25:75:0.36		51.0	2.5
1.25	25:75:0.50		50.4	2.0
2.4	25:75:0.96		49.5	1.5
10.4	25:75:4.16		47.3	1.0
